# Basic and applied biology of the primate reproductive tract – a symposium in honor of the career of Dr Robert M Brenner: Preface

**DOI:** 10.1186/1477-7827-4-S1-S1

**Published:** 2006-10-09

**Authors:** Richard L Stouffer

**Affiliations:** 1 Division of Reproductive Sciences, Oregon National Primate Research Center, Oregon Health & Science University, 505 NW 185th Avenue, Beaverton, OR 97006, USA

The following treatise includes review chapters from presentations of distinguished scientists at a symposium held August 16–17, 2005, in Portland, Oregon, entitled "Basic and applied biology of the primate reproductive tract: in Honor of the career of Dr Robert M Brenner". As a prelude to the 2005 annual meeting of the American Society of Primatologists, outstanding scientists from Europe and America assembled to present the current state-of-research in primate reproductive tract biology and to recognize Dr. Brenner's tremendous impact on this research field. In addition, numerous other peers and former students attended and contributed to the scientific exchange and social festivities. Generous support from the National Center for Research Resources (R13 RR018799) and the Oregon National Primate Research Center (ONPRC), Division of Reproductive Sciences, made this event possible.

In 2005, Bob Brenner (Figure 1) retired after 41 years as a member of the scientific staff at ONPRC, completing an exemplary career investigating basic and applied aspects of reproductive tract biology in primates. He has enjoyed continual NIH grant support since 1960 – first as a fledgling assistant professor in the Biology Department at Brown University, and then in Oregon after Bob was recruited by Director, William Montagna, to build a research program at the newly created Primate Center in 1964. Bob also succeeded in making his science, particularly his electron micrographs, immunocytochemistry and in situ hybridization, an art form. His outstanding contributions on the oviduct and uterus grace many authors' chapters, from Bloom & Fawcett's and Weiss & Greep's textbooks in histology in the 1970s, to his own seminal reviews and chapters through the 1990s. There is little wonder why Bob's expertise and collegial nature led to numerous productive collaborations with scientists around the world (e.g., Dr. Kris Chwalisz at Schering AG, Berlin and now TAP Pharmaceuticals, Chicago; Drs. David Baird and Hilary Critchley, University of Edinburgh). Bob's leadership and training accomplishments rival his science. Dr. Brenner served as Head, Division of Reproductive Biology and Behavior for 13 years from 1983 – 1996. It's because of Bob and his associates that other scientists (including myself) chose to migrate to ONPRC and attempt to keep reproductive science in the forefront of ONPRC's mission. In addition, Dr. Brenner mentored many trainees who continue to forge academic, pharmaceutical and government careers.

So, Bob, we applaud your extraordinary accomplishments during 40 great years at ONPRC! On behalf of your many colleagues and friends at the Center and around the world, we wish you the best and look forward to your continued activities as Scientist Emeritus.

## Figures and Tables

**Figure 1:**
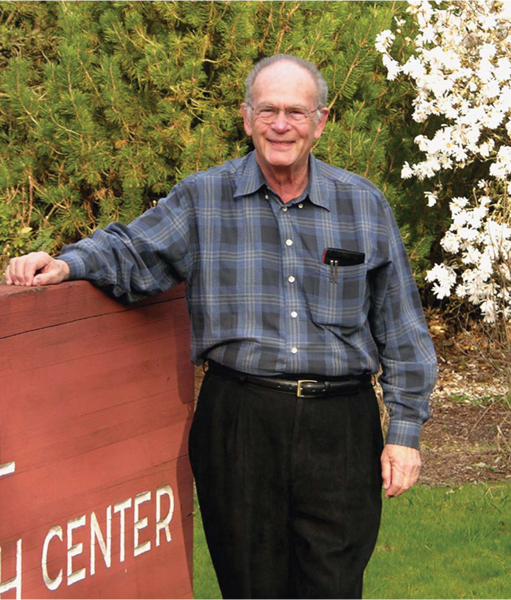
Dr Robert M Brenner.

